# Prognostic Value of HALP Score for In-Hospital Mortality in Patients with Infective Endocarditis

**DOI:** 10.3390/jcm15072707

**Published:** 2026-04-03

**Authors:** Emirhan Hancıoğlu, Sevgi Özcan, Sevil Tuğrul Yavuz, Asım Enhoş, Ertuğrul Okuyan

**Affiliations:** 1 Department of Cardiology, Istanbul Medipol University, Acıbadem Medipol District Hospital, Acıbadem, Şht. Emin Çölen Sokağı No:18, 34718 Kadıkoy, Turkey; 2 Department of Cardiology, Bağcılar Training and Research Hospital, 34200 Istanbul, Turkey; sevgibozcan@gmail.com; 3 Department of Cardiology, Başakşehir Çam and Sakura Training and Research Hospital, 34480 Istanbul, Turkey

**Keywords:** infective endocarditis, HALP score, mortality

## Abstract

**Background**: Infective endocarditis (IE) remains associated with high morbidity and mortality despite advances in diagnostic and therapeutic strategies. Markers reflecting both inflammatory burden and nutritional status may improve early risk stratification. The hemoglobin-albumin-lymphocyte-platelet (HALP) score is a composite index integrating hematologic and nutritional parameters; however, its prognostic value in IE has not been well established. **Methods**: This two-center retrospective cohort study included 218 adult patients hospitalized with IE between January 2016 and January 2025. HALP score was calculated from admission laboratory values. The primary outcome was in-hospital mortality, and 1-year mortality was evaluated as a secondary outcome. Receiver operating characteristic (ROC) analysis was used to determine the optimal cut-off value. Patients were categorized into low- and high-HALP groups, and survival was assessed using Kaplan–Meier analysis. Cox regression analyses were performed to identify independent predictors of in-hospital mortality. **Results**: A total of 218 patients were analyzed. In-hospital mortality occurred in 38.5% of patients. HALP score was significantly lower in non-survivors and was independently associated with in-hospital mortality. ROC analysis demonstrated good discriminatory performance (AUC 0.784), with an optimal cut-off value of 15.1 (sensitivity 73.9%, specificity 73.8%). Low HALP scores were associated with more advanced functional status, more frequent intracardiac complications, and higher rates of acute heart failure, renal failure, and septic shock. One-year mortality was also higher in the low-HALP group (42.9% vs. 18.2%, *p* = 0.005). **Conclusions**: HALP score is independently associated with in-hospital mortality in patients with IE and identifies a subgroup with more severe disease and worse outcomes. As an easily calculated parameter, it may serve as a complementary tool for risk stratification and clinical decision-making.

## 1. Introduction

Infective endocarditis (IE) is a severe infectious disease characterized by microbial infection of the cardiac valves and other endocardial structures, including the mural endocardium and intracardiac devices [1]. The incidence of IE has increased in recent decades, partly due to the aging population and the growing use of intracardiac devices and invasive procedures. Although advances in antimicrobial therapy, imaging modalities, and surgical management have improved diagnostic and therapeutic strategies, IE continues to be associated with high morbidity and mortality. In the literature, in-hospital mortality rates remain substantial, ranging from approximately 15% to 30% [2,3].

Inflammation and nutritional status have been closely linked to disease severity and clinical outcomes in IE [4,5]. In this context, several prognostic indices derived from routine laboratory parameters have gained increasing attention [6,7,8], as there is a need for simple, rapidly available markers for early identification of high-risk patients. The Hemoglobin, Albumin, Lymphocyte, and Platelet (HALP) score is a combined index that integrates markers of nutritional status, immune function, and hematological reserve. Initially introduced in oncology patients [9], the HALP score has subsequently been associated with prognosis in various cardiovascular conditions, including coronary artery disease, heart failure, and atrial fibrillation [10,11,12]. However, evidence regarding the prognostic significance of the HALP score in patients with IE remains limited and inconsistent in the literature.

Given the complex and dynamic course of IE, accurate risk stratification remains challenging in routine practice. In this study, we aimed to evaluate the association between the HALP score and in-hospital and 1-year mortality in patients with IE.

## 2. Methods

### 2.1. Study Design and Population

**Figure 1:**
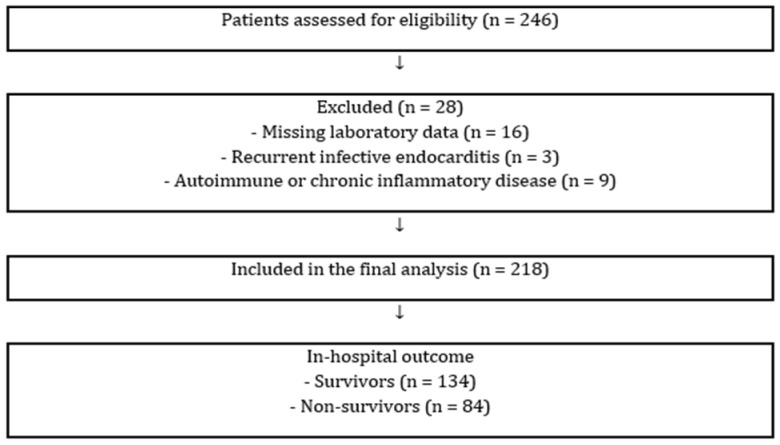
Flow diagram of patient selection.

This study was conducted as a two-center retrospective cohort study. Patients aged ≥18 years who were hospitalized with a diagnosis of IE between January 2016 and January 2025 were consecutively included. The diagnosis of IE was established according to the modified Duke criteria, based on clinical, microbiological, and echocardiographic findings [13]. The exclusion criteria were as follows: known hematological disorders; a history of organ transplantation; recurrent infective endocarditis; and chronic inflammatory or autoimmune diseases. Patients with missing laboratory data were also excluded from the study. The study population was categorized into two groups: survivor and non-survivor groups based on in-hospital mortality. The patient selection process is shown in Figure 1. The study protocol was approved by the local ethics committee, and the requirement for informed consent was waived due to the retrospective nature of the study.

### 2.2. Data Collection and Clinical Management

Demographic, clinical, and laboratory data were collected from the local hospital electronic database and patient records. Blood samples for routine laboratory analyses were collected within the first 24 h of admission. Blood cultures were obtained, with at least two sets collected from separate venipuncture sites prior to the initiation of antimicrobial therapy whenever feasible. Additional laboratory and clinical variables, including inflammatory markers, renal function parameters, and microbiological findings from blood cultures, were recorded for descriptive and prognostic analyses. IE-related complications were recorded during follow-up, such as embolic events, septic complications, renal failure, acute heart failure, surgical intervention. All patients were initiated on empirical antibiotic therapy at admission, which was subsequently adjusted according to blood culture results. Decisions regarding surgical management were made by a multidisciplinary Endocarditis Team.

The HALP score was calculated using the following formula: HALP = [hemoglobin (g/dL) × albumin (g/dL) × lymphocyte count (/µL)]/platelet count (×10^9^/L). In addition to the HALP score, other inflammation- and nutrition-based indices, including the systemic immune-inflammation index (SII), prognostic inflammatory value (PIV), prognostic nutritional index (PNI), and C-reactive protein to albumin ratio (CAR), were also calculated using standard formulas described in the previous studies [6,7,14].

All patients initially underwent transthoracic echocardiography, followed by transesophageal echocardiography for further detailed evaluation when clinically indicated. Examinations were performed using an iE33 ×MATRIX Echocardiography System (Philips, Eindhoven, The Netherlands). The assessment included evaluation of valvular involvement and valve type (native or prosthetic valves and affected valve location), presence and characteristics of vegetations, left ventricular systolic function, and intracardiac complications related to IE, including perivalvular abscess formation, leaflet perforation, paravalvular regurgitation, chordae tendineae rupture, and prosthetic valve dehiscence. All findings were systematically recorded for analysis.

### 2.3. Outcomes

The primary outcome of the study was in-hospital mortality, defined as death occurring during the index hospitalization. In addition, 1-year all-cause mortality was also evaluated based on available follow-up data obtained from hospital records. Mortality outcomes were compared between low and high HALP score groups.

### 2.4. Statistical Analysis

Statistical analyses were carried out using IBM SPSS Statistics for Windows, version 26.0 (IBM Corp., Armonk, NY, USA). Continuous variables were summarized as mean ± standard deviation (SD), and categorical variables as number and percentage. Group comparisons were performed using the chi-square test for categorical data and either Student’s *t*-test or Mann–Whitney U test for continuous variables, depending on distribution characteristics. Cox regression analysis was applied to determine the relationship between in-hospital mortality and the independent predictors. Variables that showed statistical significance in the univariable analysis were integrated into the multivariable Cox regression model. To minimize the risk of overfitting, the number of variables included in the multivariable Cox regression model was limited according to the number of events. As there were 84 deaths in the cohort, not all variables presented in Table 1 were included in the model. Univariable Cox regression analyses were first performed, and variables with statistical significance were considered for the multivariable analysis. Due to the limited number of events, only significant laboratory parameters were included as candidate predictors in the final multivariable model. Multicollinearity among predictors was assessed using the Variance Inflation Factor (VIF).

The discriminative ability of the HALP score for predicting in-hospital mortality was assessed by receiver operating characteristic (ROC) curve analysis and quantified using the area under the curve (AUC). The optimal cut-off value for the HALP score was determined using the Youden index. Patients were subsequently categorized into low-HALP and high-HALP groups according to this cut-off value. Survival probabilities were estimated with the Kaplan–Meier method and compared using the log-rank test. A two-tailed *p* value < 0.05 was considered statistically significant.

## 3. Results

A total of 246 patients were initially screened. After exclusion criteria were applied, 218 patients with IE were included in the study. Of these, 134 (61.5%) survived to hospital discharge and 84 (38.5%) died during hospitalization. Baseline demographic, clinical, and laboratory characteristics of the study population according to in-hospital mortality are presented in Table 1. The mean age of the study population was 57.5 ± 15.7 years, and 59.6% of the patients were male. Non-survivors were significantly older than survivors (62.4 ± 14.8 vs. 54.4 ± 15.5 years, *p* < 0.001).

Hypertension was more frequent among non-survivors (64.3% vs. 50.7%, *p* = 0.034). BMI was lower in non-survivors compared with survivors (24.4 ± 5.2 vs. 26.4 ± 4.7 kg/m^2^, *p* = 0.005). Other comorbidities, including diabetes mellitus, chronic heart failure, coronary artery disease, atrial fibrillation, and chronic renal failure, were more common in non-survivors but did not differ significantly between groups.

Regarding hematological parameters, white blood cell count (13.9 ± 8.1 vs. 11.1 ± 6.2 × 10^3^/µL, *p* = 0.003) and neutrophil count (12.1 ± 8.2 vs. 8.1 ± 5.1 × 10^3^/µL, *p* < 0.001) were higher in non-survivors, whereas lymphocyte counts were significantly lower (1.1 ± 0.6 vs. 1.5 ± 0.7 × 10^3^/µL, *p* < 0.001). In addition, markers of systemic inflammation and cardiac injury, including C-reactive protein (129.9 ± 92.1 vs. 97.5 ± 83.9 mg/L, *p* = 0.008), procalcitonin (median 1.1 vs. 0.6 µg/L, *p* = 0.015), troponin T (median 82.7 vs. 35.4 ng/dL, *p* < 0.001), and NT-proBNP (median 6833 vs. 553 pg/mL, *p* < 0.001), were significantly higher in non-survivors. Similarly, renal function parameters differed between groups, with lower estimated glomerular filtration rate (48.1 ± 32.7 vs. 61.8 ± 40.1 mL/min/1.73 m^2^, *p* = 0.023) and higher blood urea nitrogen levels (80.4 ± 56.6 vs. 54.9 ± 45.2 mg/dL, *p* = 0.002) in non-survivors. Serum uric acid and glucose levels were also higher among non-survivors (all *p* < 0.05).

HALP score was markedly lower in non-survivors compared with survivors (14.9 ± 8.6 vs. 27.1 ± 22.1, *p* < 0.001). In addition to the HALP score, other inflammation-based indices were also significantly different between survivors and non-survivors. Non-survivors had higher systemic immune-inflammation index (SII), prognostic inflammatory value (PIV), C-reactive protein to albumin ratio (CAR), as well as lower prognostic nutritional index (PNI) compared with survivors (all *p* < 0.05).

TTE evaluation demonstrated left-sided vegetation in 153 patients (70.2%), with similar distribution between survivors and non-survivors. Right-sided vegetations were more frequently observed in survivors (23.9% vs. 11.9%, *p* = 0.021), whereas combined left–right involvement was significantly more common in non-survivors (19.0% vs. 5.2%, *p* = 0.002). Non-survivors had a markedly higher prevalence of intracardiac complications, including leaflet perforation (41.7% vs. 14.2%, *p* < 0.001) and abscess or fistula formation (21.4% vs. 9.7%, *p* = 0.014). Vegetation size ≥ 10 mm was significantly more frequent among non-survivors (83.3% vs. 66.4%, *p* = 0.004). Regarding IE type, native valve endocarditis was the most common presentation overall (57.3%), followed by prosthetic valve IE (20.6%). Device-related IE was more frequent in survivors than non-survivors (12.7% vs. 3.6%, *p* = 0.029).

Blood culture negativity was similar between survivors and non-survivors (14.2% vs. 13.1%, *p* = 0.495). *Staphylococcus aureus* was the most frequently isolated microorganism in the overall cohort (30.3%) and was more common in non-survivors than in survivors (35.7% vs. 26.9%), although the difference was not statistically significant (*p* = 0.109). The distribution of other pathogens was comparable between groups, including streptococci (10.4% vs. 7.1%), coagulase-negative staphylococci (19.4% vs. 16.7%), *Enterococcus faecalis* (8.2% vs. 8.3%), and Candida species (3.7% vs. 8.3%), with no significant differences observed. Gram-negative microorganisms were identified in 4.5% of survivors and 7.1% of non-survivors (*p* = 0.401). Other microorganisms were significantly more frequent in survivors compared with non-survivors (10.4% vs. 1.2%, *p* = 0.006).

NYHA functional class differed significantly between groups, with non-survivors more frequently classified as NYHA III–IV, whereas survivors were predominantly in NYHA class I–II (*p* < 0.001). Regarding IE-related clinical complications, acute heart failure and septic shock were significantly more frequent in non-survivors (72.6% vs. 13.4% and 48.8% vs. 2.2%, respectively; both *p* < 0.001). Embolic events were also more common among non-survivors (40.5% vs. 22.4%, *p* = 0.004). In addition, renal failure and the need for renal replacement therapy occurred more frequently in the non-survivor group (25.0% vs. 5.2%, *p* < 0.001 and 27.7% vs. 9.0%, *p* = 0.002, respectively). Surgical treatment was performed in 125 patients (57.3%) overall. The rate of surgery was significantly higher among survivors compared to non-survivors (67.9% vs. 40.5%, *p* < 0.001).

**Figure 2:**
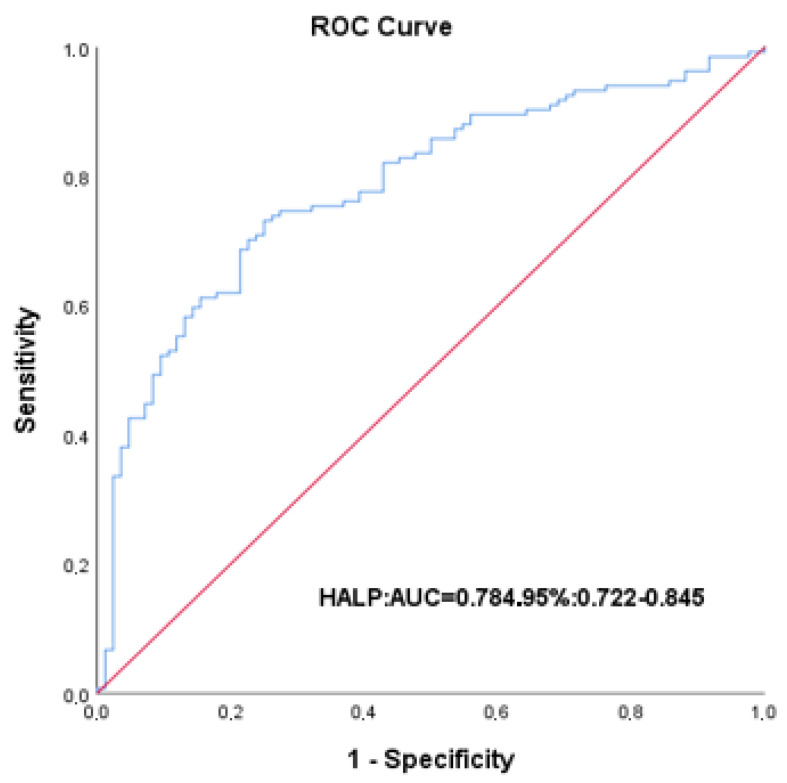
Receiver operating characteristic (ROC) curve of the HALP score for predicting in-hospital mortality in patients with infective endocarditis.

ROC curve analysis was performed to evaluate the ability of the HALP score to predict in-hospital mortality. The HALP score demonstrated good discriminatory performance, with an area under the curve of 0.784 (95% CI: 0.722–0.845, *p* < 0.001). The optimal cut-off value, determined using the Youden index, was identified as 15.1, yielding a sensitivity of 73.9% and a specificity of 73.8% (Figure 2). Based on this cut-off value, patients were stratified into low HALP (<15.1) and high HALP (≥15.1) groups (Table 2).

Demographic characteristics were similar between the low- and high-HALP groups. However, end-stage renal disease was more frequent in the low-HALP group (25.8% vs. 14.9%, *p* = 0.033). Compared with the high-HALP group, patients with low HALP scores more frequently presented with advanced NYHA class (III–IV) (*p* = 0.003). Surgical treatment was performed more frequently in the high-HALP group (62.8% vs. 50.5%, *p* = 0.046). With respect to echocardiographic findings, abscess or fistula formation (19.6% vs. 9.9%, *p* = 0.033) and leaflet perforation (37.1% vs. 14.9%, *p* < 0.001) were significantly more common in the low-HALP group. Microbiological analysis showed that *Staphylococcus aureus* infection was more common in the low-HALP group (37.1% vs. 24.8%, *p* = 0.035), whereas coagulase-negative staphylococci were more frequently observed in the high-HALP group (23.1% vs. 12.4%, *p* = 0.030). Other microorganisms were similarly distributed between the groups. Regarding clinical outcomes, patients in the low-HALP group experienced significantly higher rates of heart failure (48.5% vs. 26.4%, *p* = 0.001), renal failure (20.6% vs. 6.6%, *p* = 0.002), septic shock (37.1% vs. 6.6%, *p* < 0.001), and in-hospital renal replacement therapy (25.7% vs. 8.8%, *p* = 0.003). Importantly, in-hospital mortality was markedly higher in the low-HALP group compared with the high-HALP group (63.9% vs. 18.2%, *p* < 0.001). Similarly, 1-year mortality was significantly higher in the low-HALP group (42.9% vs. 18.2%, *p* = 0.005) (Table 2).

**Figure 3:**
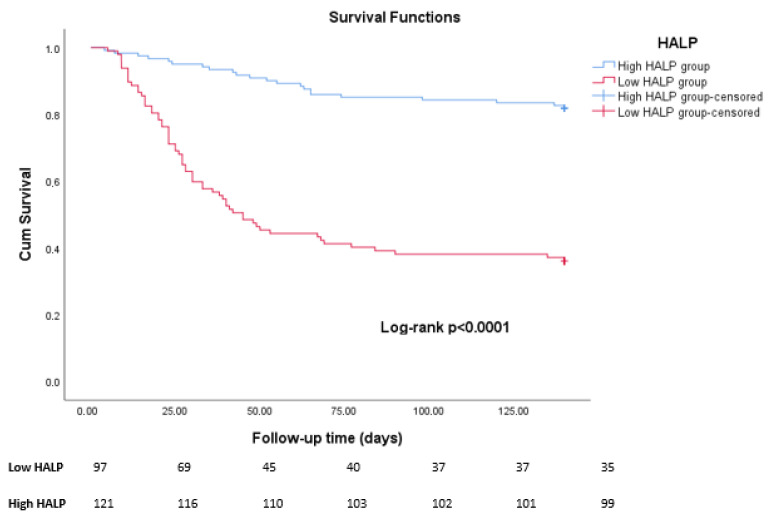
Kaplan–Meier survival curves for in-hospital mortality according to low and high HALP score groups in patients with infective endocarditis.

In univariate analysis, HALP, CAR, PNI, procalcitonin, NT-proBNP, glomerular filtration rate, pan-immune inflammation value, and systemic immune-inflammatory index were significantly associated with in-hospital mortality (all *p* < 0.05). These factors were subsequently entered into multivariate Cox regression analysis. The multivariate analysis revealed that only HALP score (HR 0.957, 95% CI 0.933–0.981, *p* = 0.001) and CAR (HR 1.089, 95% CI 1.017–1.166, *p* = 0.014) were independent predictors of in-hospital mortality (Table 3). Kaplan–Meier survival analysis showed that patients in the low HALP group had a significantly higher in-hospital mortality compared with those in the high HALP group. The survival curves separated early during follow-up and continued to diverge over time. Patients in the low-HALP group showed markedly poorer survival outcomes throughout the follow-up period (log-rank *p* < 0.001) (Figure 3).

## 4. Discussion

In this study, we investigated the prognostic value of the HALP score for in-hospital mortality in patients hospitalized with IE. Our results demonstrated that a lower HALP score was significantly associated with in-hospital mortality. In addition, higher CAR was found to be an independent predictor of in-hospital death. ROC analysis showed that the HALP score had good discriminatory ability for predicting in-hospital mortality. A cut-off value of 15.1 yielded a sensitivity of 73.9% and a specificity of 73.8%. This finding indicates that the HALP score may represent as a practical and readily available biomarker for identifying high-risk patients in clinical practice.

Despite ongoing advances in diagnostic and therapeutic strategies, patients hospitalized with IE continue to experience considerable early mortality [15]. Contemporary cohorts still report substantial in-hospital death rates, particularly among patients with advanced disease, extensive cardiac involvement, or severe systemic complications. In our study, the in-hospital mortality rate was 38.5%, consistent with findings from previous studies.

The pathophysiology of IE involves interacting mechanisms between persistent systemic inflammation, immune dysregulation, and metabolic stress. Ongoing inflammatory activation contributes to endothelial damage, vegetation progression, and organ dysfunction, while impaired nutritional status and reduced immune competence may adversely affect host defense and recovery [16,17]. In the literature, several immune and nutrition-based indices, including SII, PIV, PNI have been explored as prognostic markers in IE [18,19]. In our study, although these indices were associated with in-hospital mortality in univariate analyses, they did not retain independent prognostic significance in multivariate models. Moreover, in a recent study comparing inflammation-based prognostic indices in patients with IE, the peak CAR was identified as a superior biomarker for predicting adverse outcomes, outperforming several other inflammatory markers [20]. In line with these findings, CAR emerged as an independent predictor of in-hospital mortality in our cohort.

HALP score is derived from hemoglobin, albumin, lymphocyte count, and platelet count and reflects the combined impact of inflammatory activity and nutritional status. It was first evaluated in patients with gastric malignancies and has more recently been applied in various cardiovascular diseases [21,22]. In a study conducted by Liu et al., high HALP scores were associated with a reduced risk of 1-month and 1-year mortality in patients with heart failure [11]. In another study, Karakayali et al. found that HALP score was independently associated with in-hospital mortality in patients with ST-elevation myocardial infarction undergoing primary percutaneous coronary intervention [23]. Similarly, Koyuncu et al. demonstrated that patients undergoing coronary artery bypass grafting with low HALP scores had significantly higher one-month mortality [24]. Additionally, in a study, the HALP score was shown to be independently associated with adverse in-hospital clinical outcomes in patients undergoing transcatheter aortic valve replacement [25].

Several clinical and disease-related factors have previously been shown to be associated with increased mortality in IE [26,27]. In our cohort, when we stratified patients according to the identified HALP cut-off value, high-risk clinical features were more frequently observed in the low-HALP group, with these patients more often presenting with advanced NYHA class III-IV and end-stage renal disease. Echocardiographic evaluation was essential for identifying intracardiac complications associated with IE [28]. In our study, patients in the low-HALP group also had higher rates of abscess formation and leaflet perforation, indicating more extensive intracardiac involvement. In addition, acute heart failure, renal failure, septic shock, and the need for renal replacement therapy were significantly higher among patients with low HALP scores. Surgical treatment is associated with improved survival in complicated IE [29,30]. Notably, our study revealed that surgical treatment was more frequently performed in patients with higher HALP scores. This may reflect that patients with better overall clinical and nutritional status are more likely to be considered suitable candidates for surgical intervention. However, our study primarily aimed to evaluate the prognostic significance of the HALP score rather than the interaction between HALP and treatment strategies. Therefore, further studies are needed to determine whether HALP may influence treatment decisions or modify the effects of surgical management. The more severe clinical profile observed in the low-HALP group may partly explain the markedly higher in-hospital and 1-year mortality in these patients. The survival analysis further supported these findings. Kaplan–Meier curves demonstrated a clear separation between the two groups, with significantly lower survival rates observed in patients with low HALP scores. The survival curves diverged early during follow-up, suggesting that the HALP score may be useful for early risk stratification in patients with IE.

Each component of the HALP score has been previously linked to adverse outcomes in IE [6,31]; however, their individual prognostic impact may vary across patient populations. In our study, hemoglobin and albumin levels alone were not significantly different between survivors and non-survivors, whereas lymphocyte and platelet counts were lower in non-survivors. These findings suggest that the prognostic value of HALP may arise from the cumulative effect of multiple pathophysiological domains rather than the impact of any single laboratory parameter. By integrating hematological, inflammatory, and nutritional signals, HALP may better reflect the overall disease burden and physiological vulnerability in patients with IE.

Taken together, our findings highlight the potential clinical utility of the HALP score as a simple and readily available risk stratification tool in patients with IE. Because it is obtained from routine laboratory parameters, HALP can be easily integrated into daily clinical practice without additional cost or testing. Identification of patients with low HALP scores may help clinicians recognize a high-risk subgroup characterized by more severe disease and poorer outcomes. Early recognition of such patients may provide closer hemodynamic monitoring, timely multidisciplinary evaluation, and more individualized therapeutic decision-making, including consideration of surgical intervention when appropriate. Importantly, HALP score should be viewed as a complementary tool rather than a standalone predictor. In this context, the HALP score may provide additional prognostic insight compared with previously studied inflammatory or nutritional indices in patients with IE.

Our study has some limitations. First, its retrospective design may have introduced selection bias and limited the ability to establish causal relationships. Second, although conducted in two centers, the sample size was relatively modest, which may limit the generalizability of the findings. Third, laboratory parameters used to calculate the HALP score were obtained at admission only, and dynamic changes during hospitalization were not evaluated. Additionally, residual confounding factors cannot be completely excluded despite multivariate adjustment. Furthermore, although the HALP score was associated with mortality, we did not evaluate a formal interaction between HALP and treatment strategies such as surgical management. Therefore, whether HALP modifies the effect of surgery on clinical outcomes remains to be clarified in future studies. External validation in larger, prospective, and multicenter cohorts is warranted to confirm the prognostic utility of the HALP score in patients with IE.

## 5. Conclusions

HALP score was independently associated with in-hospital mortality in patients with IE and effectively identified a high-risk clinical phenotype. Patients with low HALP scores exhibited more severe disease and significantly higher in-hospital mortality, as well as increased 1-year mortality. As a simple and easily calculated parameter, the HALP score may serve as a valuable complementary tool for early risk stratification and individualized clinical management in this population.

## Figures and Tables

**Table 1:** Baseline demographic, clinical, and laboratory characteristics of the study population according to in-hospital mortality.

Variables	Overall n = 218	Survivors n = 134	Non-Survivors n = 84	*p*
Demographic Data and Comorbidities				
Age (y)	57.5 ± 15.7	54.4 ± 15.5	62.4 ± 14.8	<0.001
Male, n (%)	130 (59.6)	84 (62.7)	46 (54.8)	0.154
Body mass index, (kg/m^2^)	25.6 ± 5.1	26.4 ± 4.7	24.4 ± 5.2	0.005
Current smoking, n (%)	46 (21.1)	27 (20.1)	19 (22.6)	0.393
Diabetes mellitus, n (%)	82 (37.6)	45 (33.6)	37 (44.0)	0.151
Hypertension, n (%)	122 (56.0)	68 (50.7)	54 (64.3)	0.034
Previous myocardial infarction, n (%)	19 (8.7)	9 (6.7)	10 (11.9)	0.142
Chronic heart failure, n (%)	37 (17.0)	22 (16.4)	15 (17.9)	0.853
Chronic obstructive pulmonary disease, n (%)	16 (7.3)	11 (8.2)	5 (6.0)	0.534
Stroke, n (%)	45 (20.6)	23 (23.0)	22 (33.8)	0.089
Hyperlipidemia, n (%)	68 (31.2)	37 (27.6)	31 (36.9)	0.099
Coronary Artery Disease, n (%)	73 (33.5)	39 (29.1)	34 (40.5)	0.057
Atrial Fibrillation, n (%)	44 (20.2)	23 (17.2)	21 (25.3)	0.102
Chronic renal failure, n (%)	76 (34.9)	45 (33.6)	31 (36.9)	0.662
ESRD, n (%)	43 (19.7)	25 (18.7)	18 (21.4)	0.617
Malignancy history, n (%)	13 (6.0)	7 (5.2)	6 (7.1)	0.560
Previous cardiac surgery				
Previous coronary artery bypass graft, n (%)	15 (6.9)	8 (6.0)	7 (8.3)	0.664
Previous valve surgery, n (%)	48 (22.0)	28 (20.9)	20 (23.8)	
Surgery, n (%)	125 (7.3)	91 (67.9)	34 (40.5)	<0.001
NYHA class, n (%)				
I	74 (33.9)	71 (53.0)	3 (3.6)	<0.001
II	45 (20.6)	27 (20.1)	18 (21.4)	
III	64 (29.4)	24 (17.9)	40 (47.6)	
IV	35 (16.1)	12 (9.0)	23 (27.4)	
Echocardiographic Parameters				
Left ventricular ejection fraction (%)	54.9 ± 10.2	54.8 ± 10.7	55.1 ± 9.4	0.832
Pulmonary artery systolic pressure, mmHg	41.4 ± 13.8	39.5 ± 11.9	44.9 ± 16.5	0.094
Left side vegetation, n (%)	153 (70.2)	95 (70.9)	58 (69.0)	0.443
Right side vegetation, n (%)	42 (19.3)	32 (23.9)	10 (11.9)	0.021
Left-right side vegetation, n (%)	23 (10.6)	7 (5.2)	16 (19.0)	0.002
Abscess-Fistula, n (%)	31 (14.2)	13 (9.7)	18 (21.4)	0.014
Leaflet perforation, n (%)	54 (24.8)	19 (14.2)	35 (41.7)	<0.001
Pseudoaneurysm, n (%)	15 (6.9)	9 (6.7)	6 (7.1)	0.904
Paravalvular leakage, n (%)	17 (7.8)	7 (5.2)	10 (11.9)	0.073
Prosthetic valve dehiscence, n (%)	13 (6.0)	6 (4.5)	7 (8.3)	0.242
Vegetation size, n(%)				
<10 mm	59 (27.1)	45 (33.6)	14 (16.7)	0.004
≥10 mm	159 (72.9)	89 (66.4)	70 (83.3)	
Infective endocarditis type, n (%)				
Native valve IE	125 (57.3)	72 (53.7)	53 (63.1)	0.206
Prosthetic valve IE	45 (20.6)	26 (19.4)	19 (22.6)	0.343
Device-lead IE	20 (9.2)	17 (12.7)	3 (3.6)	0.029
Transvenous catheter IE	24 (11.0)	17 (12.7)	7 (8.3)	0.318
Other IE	4 (1.8)	2 (1.5)	2 (2.4)	0.634
Laboratory Findings				
White blood cell count, (10^3^/uL)	12.1 ± 7.1	11.1 ± 6.2	13.9 ± 8.1	0.003
Neutrophil, (10^3^/uL)	9.6 ± 6.8	8.1 ± 5.1	12.1 ± 8.2	<0.001
Lymphocyte, (10^3^/uL)	1.4 ± 0.7	1.5 ± 0.7	1.1 ± 0.6	<0.001
Hemoglobin, (g/dL)	9.9 ± 2.2	10.1 ± 2.3	9.7 ± 2.1	0.175
Platelet, (10^3^/uL)	227.5 ± 114.6	241.2 ± 109.8	205.5 ± 119.2	0.025
Serum C-Reactive Protein, (mg/L)	110.1 ± 88.3	97.5 ± 83.9	129.9 ± 92.1	0.008
Procalcitonin, µg/L	0.7 (0.02–36.9)	0.6 (0.02–36.9)	1.1 (0.02–33.1)	0.015
Troponin T, (ng/dL)	50.9 (3.0–3963.0)	35.4 (3.0–2102.0)	82.7 (5.5–3693.0)	<0.001
NT-ProBNP (pg/mL)	1932.5 (10.9–70,000)	553.0 (12.4–70,000)	6833 (10.9–70,000)	<0.001
Serum creatinine, (mg/dL)	1.1 (0.4–10.2)	1.0 (0.4–10.2)	1.2 (0.4–9.0)	0.098
Glomerular Filtration Rate, (mL/dk/1.73 m^2^)	56.4 ± 37.9	61.8 ± 40.1	48.1 ± 32.7	0.023
Blood Urea Nitrogen, (mg/dL)	94.9 ± 51.4	54.9 ± 45.2	80.4 ± 56.6	0.002
Albumin, (g/L)	25.8 ± 14.2	26.7 ± 14.6	24.4 ± 13.4	0.241
Thyroid Stimulating Hormone, (µlU/mL)	1.9 ± 1.8	1.7 ± 1.5	2.3 ± 2.1	0.082
Aspartate transaminase, (U/L)	24.0 (3.4–673.0)	21.0 (7.0–645.0)	30.0 (3.4–673.0)	0.069
Uric acid, (mg/dL)	6.1 ± 3.1	5.6 ± 2.2	6.7 ± 4.1	0.019
Glucose, (mg/dL)	145.3 ± 70.5	132.1 ± 56.2	165.7 ± 84.6	0.003
Sodium, (mEq/L)	133.8 ± 11.7	134.9 ± 4.5	131.9 ± 17.7	0.176
Potassium, (mEq/L)	4.4 ± 0.7	4.4 ± 0.7	4.4 ± 0.6	0.915
HALP	22.4 ± 19.1	27.1 ± 22.1	14.9 ± 8.6	<0.001
CAR	6.7 ± 4.1	5.3 ± 3.4	8.8 ± 4.3	<0.001
PNI	33.2 ± 6.4	34.8 ± 5.7	30.9 ± 6.7	<0.001
Pan-Immune Inflammation Value	1467.9 ± 1302.4	1174.9 ± 1113.9	2010.6 ± 1310.1	<0.001
Systemic Immune-Inflammatory Index	1512.1 ± 1066.2	1275.6 ± 988.5	1875.9 ± 1086.2	<0.001
Microorganism				
Blood culture negative, n (%)	30 (13.8)	19 (14.2)	11 (13.1)	0.495
Staphylococcus aureus, n (%)	66 (30.3)	36 (26.9)	30 (35.7)	0.109
Streptococci, n (%)	20 (9.2)	14 (10.4)	6 (7.1)	0.284
Coagulase negative staphylococcus, n (%)	40 (18.8)	26 (19.4)	14 (16.7)	0.375
Brucella, n (%)	5 (2.3)	3 (2.2)	2 (2.4)	0.946
Candida, n (%)	12 (5.5)	5 (3.7)	7 (8.3)	0.147
Enterococcus faecalis, n (%)	18 (8.3)	11 (8.2)	7 (8.3)	0.581
Gram-negative, n (%)	12 (5.5)	6 (4.5)	6 (7.1)	0.401
Others, n (%)	15 (6.9)	14 (10.4)	1 (1.2)	0.006
Clinical complications				
Heart failure, n (%)	79 (36.2)	18 (13.4)	61 (72.6)	<0.001
Renal failure, n (%)	28 (12.8)	7 (5.2)	21 (25.0)	<0.001
Cerebrovascular accident, n (%)	47 (21.6)	20 (14.9)	27 (32.1)	0.002
Septic emboli, n (%)	21 (9.6)	11 (8.2)	10 (11.9)	0.251
Total embolic event, n (%)	64 (29.4)	30 (22.4)	34 (40.5)	0.004
Septic shock, n (%)	44 (20.2)	3 (2.2)	41 (48.8)	<0.001
Ventricular arrhythmia, n (%)	4 (1.8)	2 (1.5)	2 (2.4)	0.654
Hospital follow-up	38.8 ± 23.8	39.9 ± 19.9	36.9 ± 28.8	0.353
In-hospital renal replacement therapy, n (%)	27 (12.4)	9 (9.0)	18 (27.7)	0.002

CAR, C-reactive protein-to-albumin ratio; ESRD, End-stage renal disease; HALP, Hemoglobin-albumin-lymphocyte-platelet; NT-proBNP, N-terminal pro–B-type natriuretic peptide; NYHA, New York Heart Association; PNI, Prognostic nutritional index.

**Table 2:** Comparison of demographic, clinical, echocardiographic characteristics and outcomes between low- and high-HALP groups.

Variables	Low-HALP Group n = 97	High-HALP Group n = 121	*p*
Demographic Data and Comorbidities			
Age (y)	58.2 ± 15.9	56.9 ± 15.6	0.553
Male, n (%)	56 (57.7)	74 (61.2)	0.354
Body mass index, (kg/m^2^)	24.9 ± 4.9	26.2 ± 5.1	0.062
Current smoking, n (%)	19 (19.6)	27 (22.3)	0.375
Diabetes mellitus, n (%)	37 (38.1)	45 (37.2)	0.498
Hypertension, n (%)	57 (58.8)	65 (53.7)	0.272
Previous myocardial infarction, n (%)	9 (9.3)	10 (8.3)	0.488
Chronic heart failure, n (%)	16 (16.5)	21 (17.4)	0.507
Chronic obstructive pulmonary disease, n (%)	6 (6.2)	10 (8.3)	0.377
Stroke, n (%)	22 (29.7)	23 (25.3)	0.321
Hyperlipidemia, n (%)	33 (34.0)	35 (28.9)	0.254
Coronary Artery Disease, n (%)	33 (34.0)	40 (33.1)	0.497
Atrial Fibrillation, n (%)	23 (23.7)	21 (17.5)	0.168
Chronic renal failure, n (%)	39 (40.2)	37 (30.6)	0.090
ESRD, n (%)	25 (25.8)	18 (14.9)	0.033
Malignancy history, n (%)	8 (8.2)	5 (4.1)	0.162
Surgery, n (%)	49 (50.5)	76 (62.8)	0.046
Previous coronary artery bypass graft, n (%)	6 (6.2)	9 (7.4)	0.658
Previous valve surgery, n (%)	19 (19.6)	29 (24.0)	
NYHA class, n(%)			
I	22 (22.7)	52 (43.0)	0.003
II	21 (21.6)	24 (19.8)	
III	31 (32.0)	33 (27.3)	
IV	23 (23.7)	12 (9.9)	
Echocardiographic Parameters			
Left ventricular ejection fraction (%)	55.6 ± 9.8	54.4 ± 10.4	0.391
Pulmonary artery systolic pressure, mmHg	42.9 ± 14.7	40.2 ± 13.2	0.384
Left side vegetation, n (%)	67 (69.1)	86 (71.1)	0.431
Right side vegetation, n (%)	19 (19.6)	23 (19.0)	0.524
Left-right side vegetation, n (%)	11 (11.3)	12 (9.9)	0.451
Abscess-Fistula, n (%)	19 (19.6)	12 (9.9)	0.033
Leaflet perforation, n (%)	36 (37.1)	18 (14.9)	<0.001
Pseudoaneurysm, n (%)	8 (8.2)	7 (5.8)	0.326
Paravalvular leakage, n (%)	6 (6.2)	11 (9.1)	0.297
Prosthetic valve dehiscence, n (%)	5 (5.2)	8 (6.6)	0.439
Vegetation size, n(%)			
<10 mm	22 (22.7)	37 (30.6)	0.125
≥10 mm	75 (77.3)	84 (69.4)	
Infective endocarditis type, n(%)			
Native valve IE	58 (59.8)	67 (55.4)	0.302
Prosthetic valve IE	17 (17.5)	28 (23.1)	0.198
Device-lead IE	6 (6.2)	14 (11.6)	0.128
Transvenous catheter IE	13 (13.4)	11 (9.1)	0.213
Other IE	3 (3.1)	1 (0.8)	0.215
Microorganism			
Blood culture negative, n (%)	14 (14.4)	16 (13.2)	0.474
Staphylococcus aureus, n (%)	36 (37.1)	30 (24.8)	0.035
Streptococci, n (%)	8 (8.2)	12 (9.9)	0.428
Coagulase negative staphylococcus, n (%)	12 (12.4)	28 (23.1)	0.030
Brucella, n (%)	2 (2.1)	3 (2.5)	0.838
Candida, n (%)	7 (7.2)	5 (4.1)	0.243
Enterococcus faecalis, n (%)	6 (6.2)	12 (9.9)	0.229
Gram-negative, n (%)	6 (6.2)	6 (5.0)	0.458
Others, n (%)	6 (6.2)	9 (7.4)	0.466
Clinical complications			
Heart failure, n (%)	47 (48.5)	32 (26.4)	0.001
Renal failure, n (%)	20 (20.6)	8 (6.6)	0.002
Cerebrovascular accident, n (%)	23 (23.7)	24 (19.8)	0.299
Septic emboli, n (%)	11 (11.3)	10 (8.3)	0.295
Total embolic event, n (%)	32 (33.0)	32 (26.4)	0.183
Septic shock, n (%)	36 (37.1)	8 (6.6)	<0.001
Ventricular arrhythmia, n (%)	1 (1.0)	3 (2.5)	0.428
Hospital follow-up	36.7 ± 25.3	40.4 ± 22.4	0.249
In-hospital renal replacement therapy, n (%)	19 (25.7)	8 (8.8)	0.003
In-hospital mortality, n (%)	62 (63.9)	22 (18.2)	<0.001
1-year mortality, n (%)	15 (42.9)	18 (18.2)	0.005

ESRD, End-stage renal disease; NYHA, New York Heart Association.

**Table 3:** Univariate and multivariate Cox regression analyses for predictors of in-hospital mortality.

	Univariate HR	%95 CI	*p*	Multivariate HR	%95 CI	*p*
Serum C-Reactive Protein	0.782	0.856–1.286	0.166			
Procalcitonin	1.033	1.007–1.061	0.014	0.970	0.924–1.019	0.226
Troponin T	1.000	1.000–1.001	0.067			
NT-ProBNP	1.102	1.005–1.217	0.019	0.996	0.990–1.012	0.392
Glomerular Filtration Rate	0.992	0.986–0.997	0.004	0.995	0.989–1.002	0.155
Uric acid	1.050	1.000–1.104	0.051			
HALP	0.895	0.871–0.919	0.001	0.957	0.933–0.981	0.001
CAR	1.137	1.081–1.196	0.001	1.089	1.017–1.166	0.014
PNI	0.947	0.914–0.981	0.003	0.995	0.955–1.036	0.804
Pan-Immune Inflammation Value	1.005	1.001–1.012	0.001	1.015	0.993–1.028	0.052
Systemic Immune-Inflammatory Index	1.023	1.007–1.121	0.006	1.002	0.998–1.007	0.454

CAR, C-reactive protein-to-albumin ratio; HALP, Hemoglobin-albumin-lymphocyte-platelet score; PNI, Prognostic nutritional index.

## Data Availability

The datasets used and/or analyzed during the current study are available from the corresponding author on reasonable request.
